# 2D, 3D-QSAR study and docking of vascular endothelial growth factor receptor 3 (VEGFR3) inhibitors for potential treatment of retinoblastoma

**DOI:** 10.3389/fphar.2023.1177282

**Published:** 2023-04-07

**Authors:** Rui Ren, Liyu Gao, Guoqi Li, Shuqiang Wang, Yangzhong Zhao, Haitong Wang, Jianwei Liu

**Affiliations:** ^1^ Affiliated Hospital of Weifang Medical University, School of Clinical Medicine, Weifang Medical University, Weifang, China; ^2^ Shouguang Guangming Hospital, Shouguang, China; ^3^ Qingzhou Huiming Eye Hospital, Qingzhou, China; ^4^ Affiliated Hospital of Weifang Medical University, Weifang, China

**Keywords:** VEGFR3 inhibitor, Retinoblastoma, quantitative structure-activity relationship, heuristic method, gene expression programming

## Abstract

**Background:** Retinoblastoma is currently the most common malignant tumor seen in newborns and children’s eyes worldwide, posing a life-threatening hazard. Chemotherapy is an integral part of retinoblastoma treatment. However, the chemotherapeutic agents used in clinics often lead to drug resistance. Thus there is a need to investigate new chemotherapy-targeted agents. VEGFR3 inhibitors are anti-tumour-growth and could be used to develop novel retinoblastoma-targeted agents.

**Objective:** To predict drug activity, discover influencing factors and design new drugs by building 2D, 3D-QSAR models.

**Method:** First, linear and non-linear QSAR models were built using heuristic methods and gene expression programming (GEP). The comparative molecular similarity indices analysis (COMISA) was then used to construct 3D-QSAR models through the SYBYL software. New drugs were designed by changing drug activity factors in both models, and molecular docking experiments were performed.

**Result:** The best linear model created using HM had an R^2^, S^2^, and R^2^cv of 0.82, 0.02, and 0.77, respectively. For the training and test sets, the best non-linear model created using GEP had correlation coefficients of 0.83 and 0.72 with mean errors of 0.02 and 0.04. The 3D model designed using SYBYL passed external validation due to its high Q^2^ (0.503), R^2^ (0.805), and F-value (76.52), as well as its low standard error of SEE value (0.172). This demonstrates the model’s reliability and excellent predictive ability. Based on the molecular descriptors of the 2D model and the contour plots of the 3D model, we designed 100 new compounds using the best active compound 14 as a template. We performed activity prediction and molecular docking experiments on them, in which compound 14.d performed best regarding combined drug activity and docking ability.

**Conclusion:** The non-linear model created using GEP was more stable and had a more substantial predictive power than the linear model built using the heuristic technique (HM). The compound 14.d designed in this experiment has the potential for anti-retinoblastoma treatment, which provides new design ideas and directions for retinoblastoma-targeted drugs.

## 1 Introduction

Retinoblastoma, common among children under 3, is an intraocular malignant tumour originating from the retina. The incidence of retinoblastoma accounts for 4% of all pediatric malignant tumours (Dhami et al., 2017), ranking second among them. It can affect one or both eyes and is generally fatal to young children due to its propensity for intracranial and distant metastases. Systemic chemotherapy is the most commonly used method to prevent tumour metastasis. The most often prescribed retinoblastoma chemotherapy medicines are vincristine, etoposide, and carboplatin.

Nevertheless, when vincristine, etoposide, or carboplatin are used for an extended period, patients may have drug resistance, limiting their therapeutic usage. It is essential to research novel chemotherapeutic medicines for the treatment of retinoblastoma. Accumulating evidence suggests that hypoxia, tumour angiogenesis and the degradation of the extracellular matrix into tumour invasion and metastasis are vital factors. (Luan and Si, 2022). Vascular endothelial growth factor (VEGF), one known angiogenic factor, is vital in initiating and developing malignant tumours (Wu et al., 2018). VEGF-C/VEGFR3 is the most potent pair of lymphangiogenic regulators in tumours. Moreover, lymphatic vessels generated by this regulatory system are the morphological basis for migrating tumour cells to distant sites and their adhesion, infiltration, and metastasis to distant lymph nodes (ZEIDMAN et al., 1955; Hamada et al., 2000). To achieve the goal of retinoblastoma therapy, we can either directly inhibit tumor growth or reduce the expression of VEGFR3 to prevent the development of lymphatic and blood vessels.

It is necessary to guarantee the high selectivity and efficiency of VEGFR3 inhibitors for successful application in therapy (Jiang et al., 2019; Jiang et al., 2021). The most effective method for overcoming severe adverse effects is a selective anti-VEGFR3 treatment. (Ravi et al., 2016; Melincovici et al., 2018). Relevant studies have shown that VEFGR3 inhibitors effectively slow tumor development and lower the risk of metastasis. These VEGFR3 inhibitors have various chemical characteristics. Among them, MAZ51 is an Indometone molecule that inhibits VEGFR3 activation by inhibiting the VEGF-C-VEGFR3 pathway but does not inhibit VEGFR2 activation caused by VEGF-C. *In vivo* and *in vitro* experiments, MAZ51 mediated the apoptosis of various tumour cells, expressing anti-cell proliferation activity (Kirkin et al., 2004). Alam et al. (2012) discovered SAR131675, a selective VEGFR3 inhibitor with an IC50 of 23 nM. *In vivo* experiments suggest that SAR131675 reduced lymph node metastases and invasion of the lung node. In addition, Chang et al. (2014) identified two peptides associated with VEGFR3 that can regulate biological activity, which can selectively inhibit VEGFR3 expression and VEGF-C-pathway-mediated invasive metastasis of cancerous cells.

Li et al. used *in vitro*, *in silico*, and structure-based drug design to discover a new peptide CP-7. Other studies have shown that CP-7 is minimally toxic, highly selective for VEGFR-3, and strongly inhibits VEGFR-3-positive cancer cells *in vitro* and *in vivo* (Li et al., 2017). Currently, the FDA has authorized no small molecules that are selective for VEGFR3. We are keen to discover a novel selective VEGFR3 inhibitor with low adverse effects to treat retinoblastoma as an anti-cancer drug. According to the CruM-Brown equation, organic molecules’ nature, structure, and activity are closely related. We can look into a compound’s structure to get ideas for new medications.

In quantitative structure-activity relationship (QSAR), theoretical chemical methods are combined with various mathematical and statistical analysis techniques to quantitatively describe and study the relationships between structures and properties (Cherkasov et al., 2014). QSAR has enabled computer-aided drug design, a synthesis based on several structures and accompanying chemical data, to become a fully established and expanding field of research (Santos-Filho and Hopfinger, 2001). The QSAR model could be used to reasonably predict the inhibitory activity of VEGFR3 inhibitors while exhibiting a comparatively high accuracy. QSAR is divided into 2D and 3D methods (Roy et al., 2015). Through 2D-QSAR, the effect of molecular distribution in 3D spaces on drug activity cannot be considered. In contrast, through 3D-QSAR, the effect of 2D descriptors on drug activity cannot be considered. So, our experiments adopt a combined 2D and 3D model to predict drugs targeting the retinoblastoma VEGFR3 receptor and optimization.

## 2 Experimental

### 2.1 Data collection and its division

In this study, we used GEP to construct a non-linear QSAR model and HM to construct a linear QSAR model. Fifty compounds were obtained from the literature (Li et al., 2021), whose IC50 values are shown in Table 1. Forty compounds were randomly chosen as the training and 10 as the test sets to limit the error caused by confounding factors. This study used a small sample size. The test set was used to determine whether the model was valid after modelling, parameter adjustment, and variable selection were made on the training set.

**TABLE 1:** VEGFR3 kinase inhibition and activity values of compounds 1- 50.

Structure	R1	R2	R3	IC_50_(µM)	NO
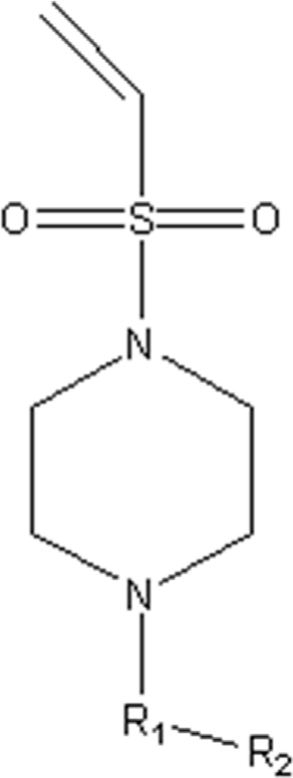	4,6-dimethylthieno[2,3-d]pyrimidine	toluene	—	15.41	1
	3,5-dimethylpyrazolo[1,5-a]pyrimidine		—	20.35	2*
	3,5-dimethylpyrazolo[1,5-a]pyrimidine	4-p-tolylmorpholine	—	17.85	3
	4,6-dimethylquinazoline	toluene	—	18.98	4
	1,7-dimethylnaphthalene		—	18.0	5*
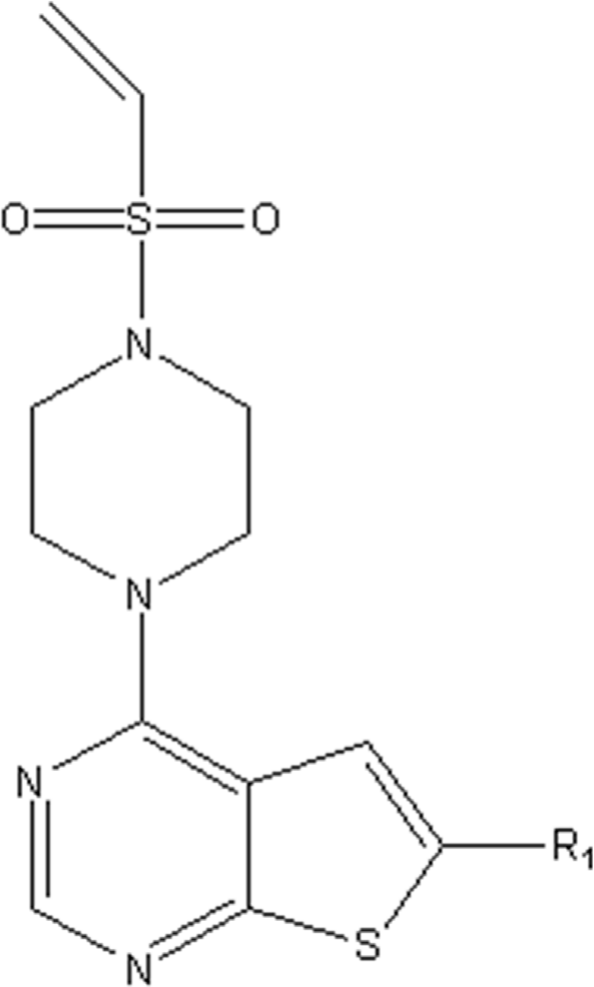	1-(trifluoromethyl)-4-methylbenzene	—	—	6.41	6*
	1-methoxy-4-methylbenzene	—	—	29.87	7
	4-methylbenzonitrile	—	—	8.05	8
	4-p-tolylmorpholine	—	—	18.38	9
	1-methyl-4-p-tolylpiperazine	—	—	7.55	10
	(4-methylpiperazin-1-yl)(p-tolyl)methanone	—	—	8.65	11*
	1-methylnaphthalene	—	—	23.65	12
	5-methylquinoline	—	—	20.97	13
	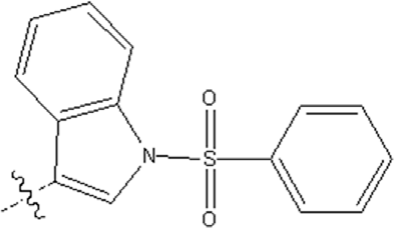	—	—	0.76	14
	2-chloro-4-methylpyridine	—	—	27.74	15*
	2-chloro-4-methylpyridine	—	—	9.27	16
	2-fluoro-5-methylpyridine	—	—	16.02	17*
	5-bromo-2-fluoro-3-methylpyridine	—	—	24.11	18
	1,3,4-trimethyl-1H-pyrazole	—	—	10.42	19*
	1-benzyl-4-methyl-1H-pyrazole	—	—	19.11	20
	1-benzyl-4-methyl-1H-pyrazole	—	—	28.17	21*
	2-methylthiophene	—	—	34.12	22
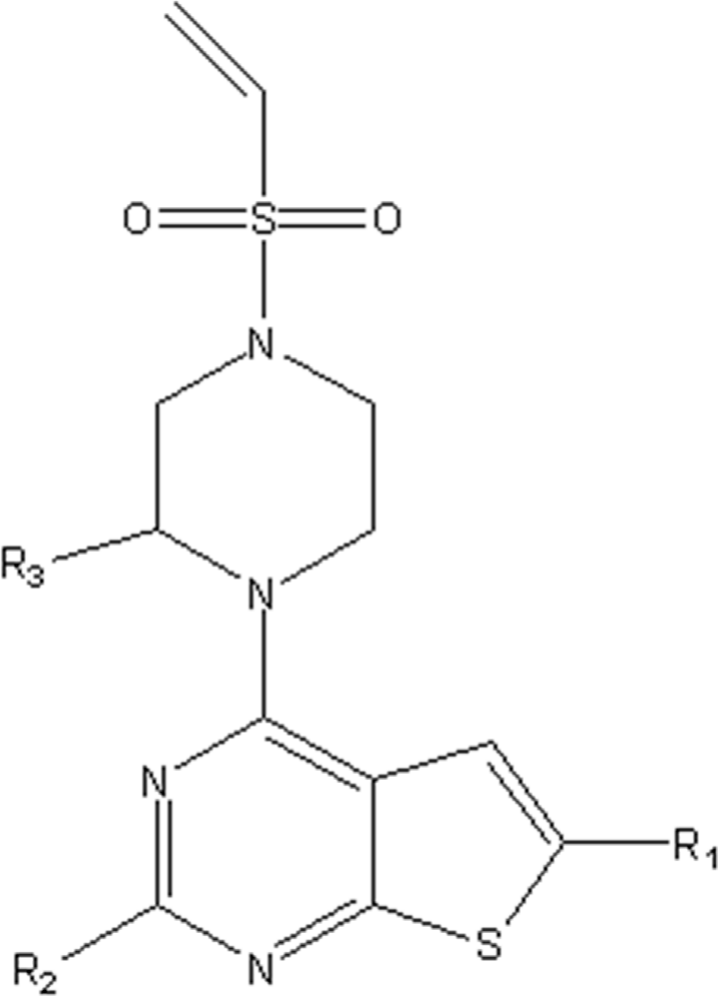	4-methylbenzonitrile	(R)-2-(methoxymethyl)-1-methylpyrrolidine	H	3.01	23
		2-(methoxymethyl)-1-methylpiperidine	H	3.18	24*
	(4-methylpiperazin-1-yl)(p-tolyl)methanone	(R)-2-(methoxymethyl)-1-methylpyrrolidine	H	4.20	25
			ethane	3.33	26
	2-methoxy-5-methylpyridine		H	3.56	27
	1-methyl-4-p-tolylpiperazine	(R)-2-(methoxymethyl)-1-methylpyrrolidine	H	40.42	28
			ethane	2.88	29
		H		6.18	30
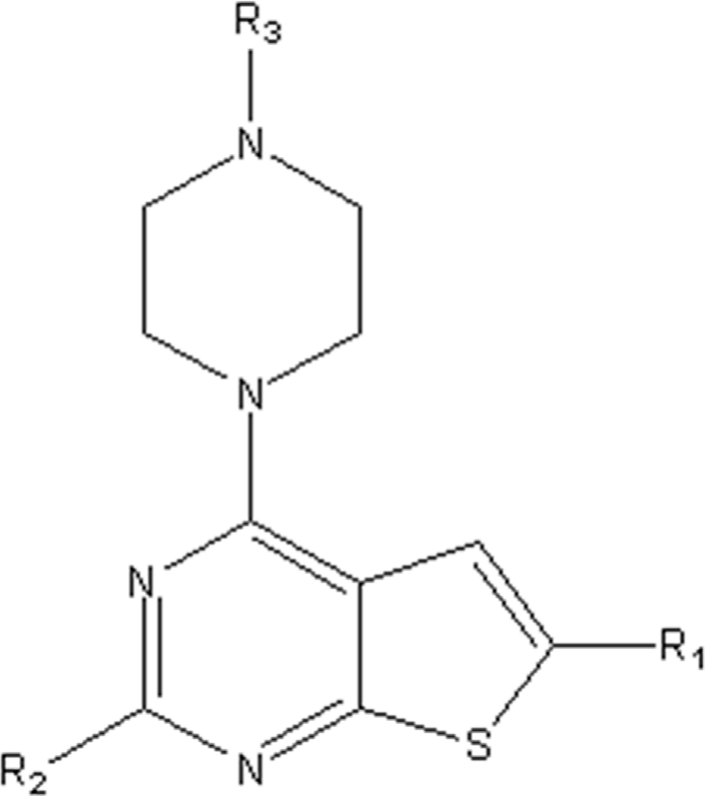	1-methyl-4-p-tolylpiperazine	H	1-((E)-2-(methylsulfonyl)vinyl)benzene	5.9	31
		H	(methylsulfonyl)ethane	7.33	32
		H	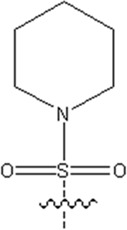	8.13	33
		H	(methylsulfonyl)cyclopropane	11.48	34
		H	N-ethylacetamide	8.33	35
		H	N-phenylacetamide	7.59	36
		H	N-(4-methoxyphenyl)acetamide	20.92	37
		H	N-(4-cyanophenyl)acetamide	3.93	38
		H	N-(4-chlorophenyl)acetamide	5.44	39
		H	N-(4-(trifluoromethoxy)phenyl)acetamide	4.60	40*
		H	N-(2-fluorophenyl)acetamide	10.27	41
		H	N-(3-fluorophenyl)acetamide	13.23	42
		H	N-(2-methoxyphenyl)acetamide	23.23	43
		H	N-(2,4-difluorophenyl)acetamide	11.43	44
		H	N-(4-chloro-3-(trifluoromethyl)phenyl)acetamide	2.22	45
		H	N-(2,6-diisopropylphenyl)acetamide	8.43	46
		H	N-(2-fluoro-5-(trifluoromethyl)phenyl)acetamide	9.09	47
	(4-methylpiperazin-1-yl)(p-tolyl)methanone	(R)-2-(methoxymethyl)-1-methylpyrrolidine	(methylsulfonyl)ethane	4.20	48
			but-3-en-2-one	5.39	49
	1-methyl-4-p-tolylpiperazine	2-(methoxymethyl)-1-methylpiperidine	1-(methylsulfonyl)benzene	4.42	50

***Note:*** * represents the test set in the 2D-QSAR experiment, and the underline represents the test set in 3D-QSAR.

### 2.2 2D-QSAR research

#### 2.2.1 Calculation of the descriptors

Chemdraw software was used to depict the two-dimensional structure of the molecule. All compounds were initially optimized using the molecular mechanical method MM + through the Hyperchem (HyperChem. 4.0, 1994) software. Using the semi-empirical PM3 method (Li et al., 2021) as a guide, the lowest-energy structure was obtained *via* geometric optimization. The molecular structure was optimized using the MOPAC 7.0 program calculation. The MOPAC file’s output was then transferred to the CODESSA program calculation in five different types: structure descriptors, topology descriptors, geometrical descriptors, static descriptors, and quantum chemical descriptors. Six hundred thirty-two descriptors in all were obtained.

#### 2.2.2 Linear model through heuristic method

2D-QSAR was used to quantify the correlation between the chemical structure and physiological activity of the molecule based on its structural properties. One of the leading research methods of 2D-QSAR is the heuristic method, which can be applied to the computer-aided design of new drugs. The CODESSA-based heuristic approach may be used to quickly create the best multivariable linear predictor of drug activity after screening many computed molecular descriptors. Identifying physicochemical factors that influence pharmacological action may also be possible and provide ideas for future drug development. The steps to build a linear model through HM were: parameter descriptors were selected according to the value of R^2^, F-test, *t*-test, and R^2^cv. First, the two-parameter correlation coefficient with the best statistical effect was determined. Second, the addition of descriptors not in use during the previous selection. The above was repeated until the maximum number of parameters obtained through the correlating equation was obtained. A linear model was developed based on HM, containing five descriptors. Cao and Lin (2003).

#### 2.2.3 Non-linear model through GEP

Portuguese scientist *Candida* Ferreira published the novel genetic algorithm, Gene Expression Programming (GEP), which has a high genotypic and phenotypic division, in 2001 (Algorithms, 1992). Genetic algorithms (GAs) and genetic programming algorithms (GPs) have evolved into GEP algorithms (Holland, 1992). However, GEP is a fundamentally-different type of individual from those used by GAs and GPs. In GAs, an individual is represented by a fixed linear string (chromosome). The individuals in GPs are split trees, non-linear entities with different lengths and shapes. Through GEP, the ease of use and simplicity of GAs are combined with the ability of GPs to find expressions. In GEP, individuals are encoded into linear strings of a fixed length (genomes or chromosomes), which are then expressed as non-linear entities with different lengths and shapes (expression trees). The algorithms enable the optimal answer to be discovered following many functions. The most remarkable feature of GEP algorithms is the ability to tackle complex problems using simple coding (Kaydani et al., 2014a; Zhang et al., 2018).

**FIGURE 1:**
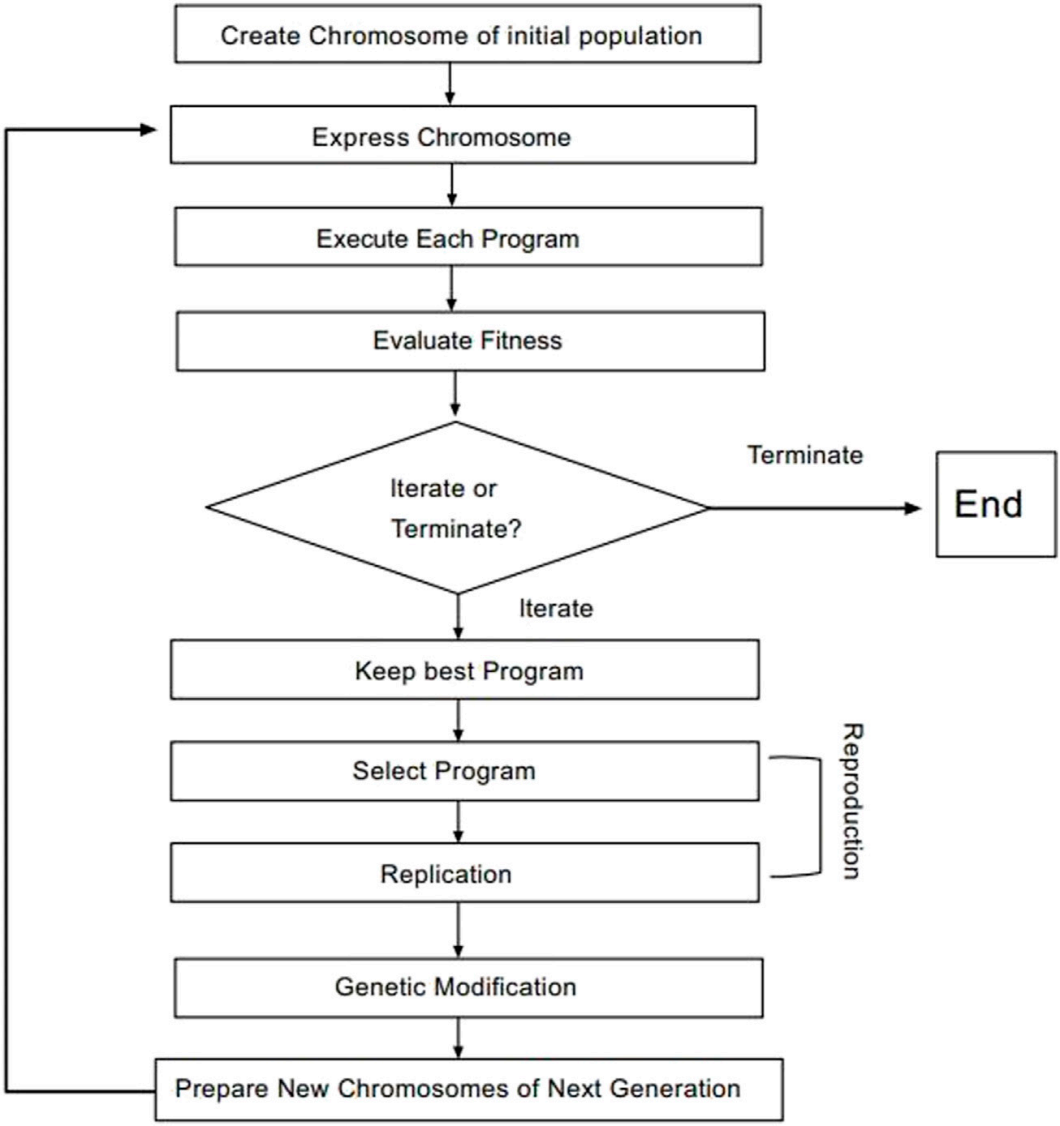
GEP flow chart.

The detailed steps of the GEP algorithms are as follows:

After being generated randomly, the initialized chromosomes were permuted into expression trees. Then, the fitness function could be used to check whether the solution meets the termination criteria. Those who didn't fit the termination criteria were retained using an elite roulette selection procedure. Selected individuals were genetically manipulated according to specific probabilistic rules for mutation, recombination, and transposition to form new individuals. In the end, a new generation was born (Teodorescu and Sherwood, 2008; Gharagheizi et al., 2012; Pham and Karaboga, 2012; Kaydani et al., 2014b). The above process is shown in Figure 1.

Considering the limitation that the effect of 3D structure on drug activity cannot be taken into account using 2D-QSAR, we must perform 3D-QSAR model construction.

### 2.3 3D-QSAR research

#### 2.3.1 Structural optimization and data grouping

The 50 compounds optimized for the subsequent 3D-QSAR experiment were drawn using ChemDraw software for the 2D-QSAR experiment. The lowest energy conformation of the compounds was searched through a systematic search method and then optimized through conjugate gradient minimization in the Triops force field. Ultimately, 3D-QSAR studies were conducted using the optimized conformation as the base conformation (Yu et al., 2015).

Similar to the 2D-QSAR experiment, which served the same function as the prior experiments, we had to separate these optimized molecules into training and test sets. We transformed the IC50 values using the -log (IC50) +6 algorithm to reduce the skewed data distribution’s adverse effects and stabilize the variance.

#### 2.3.2 Conformational sampling and alignment

**FIGURE 2:**
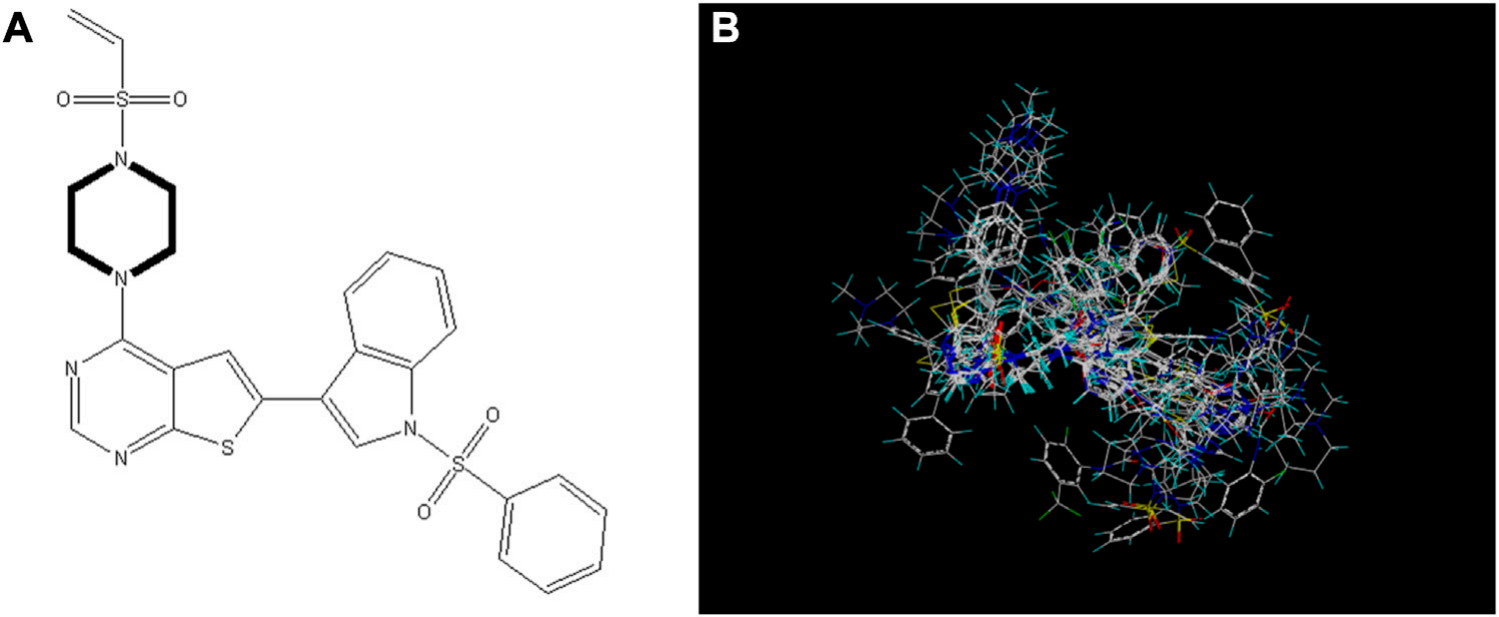
Compound 14 is used as a template for all compound alignment. **(A)** The commonly used alignment structure in compound 14 (shown in bold), **(B)** all compounds are arranged with 14 as a template.

Selecting an appropriate compound structure is crucial since building 3D-QSAR models is directly related to the compound structure (Li et al., 2005; Patel et al., 2008; Ai et al., 2011). Figure 2 shows the superimposed maps of all compounds used in this study, which involved superimposing the compound structures using the ligand comparison method. Compound 14 was utilized as the superposition template in this study because it had the best activity and standard structure.

#### 2.3.3 CoMSIA study

One of the 3D-QSAR research techniques is the comparative molecular similarity indices analysis (CoMSIA), which, using known biological activity, may assist in developing new drugs and be used to predict their biological activity (Cramer et al., 1988; Klebe et al., 1994). To obtain molecular field data, comparative molecular field analysis (CoMFA) superimposes molecules with the same structural parent ring in space so that their spatial orientations are uniform. Then it transmits a probe particle to travel around the molecule, calculates their interaction, and records the energy values of their interaction in different spatial coordinates (Chen et al., 2022a). A distance-dependent Gaussian functional form is used to calculate the interaction between probe atoms or groups and molecules through CoMSIA, which can effectively avoid the defects caused by the functional form of electrostatic and steric fields using the conventional COMFA method. The steric (S), electrostatic (E), hydrophobic (H), hydrogen bond donor (D), and hydrogen bond acceptor (A) fields are used to define five molecular field characteristics using the CoMSIA method, which clearly shows that activity is influenced by spatial, electrostatic, hydrophobic, and hydrogen bonding factors (Ajala and Okoro, 2011). In general, CoMSIA generates a 3D QSAR model that is more satisfactory.

#### 2.3.4 Validation of 3D-QSAR model

Generally, higher Q^2^, R^2^, and F values and lower SEE values can be considered models with excellent fitting ability. However, the predictiveness of the proposed model cannot be proved entirely only with these statistical parameters. Other methods are needed for further validation (Yan et al., 2020). In this experiment, we used the external validation method for validation with the following equation:

$$R_{ext}^{2}=1-\frac{\sum_{i=1}^{ntest}{\left(y_{i}-{\tilde{y}}_{i}\right)}^{2}}{{\left.\sum_{i=1}^{ntest}\left(y_{i}\right.-{\tilde{y}}_{tr}\right)}^{2}}$$

In this formula, ntest refers to the number of compounds in the test set, refers to the average value of compound activity in the training set, and, refer to the experimental and predicted values of compound activity in the test set, respectively. Typically, only when >0.5 can demonstrate that the model is stable with excellent predictive power (Yang et al., 2011; Mouchlis et al., 2012).

## 3 Results

### 3.1 HM

**FIGURE 3:**
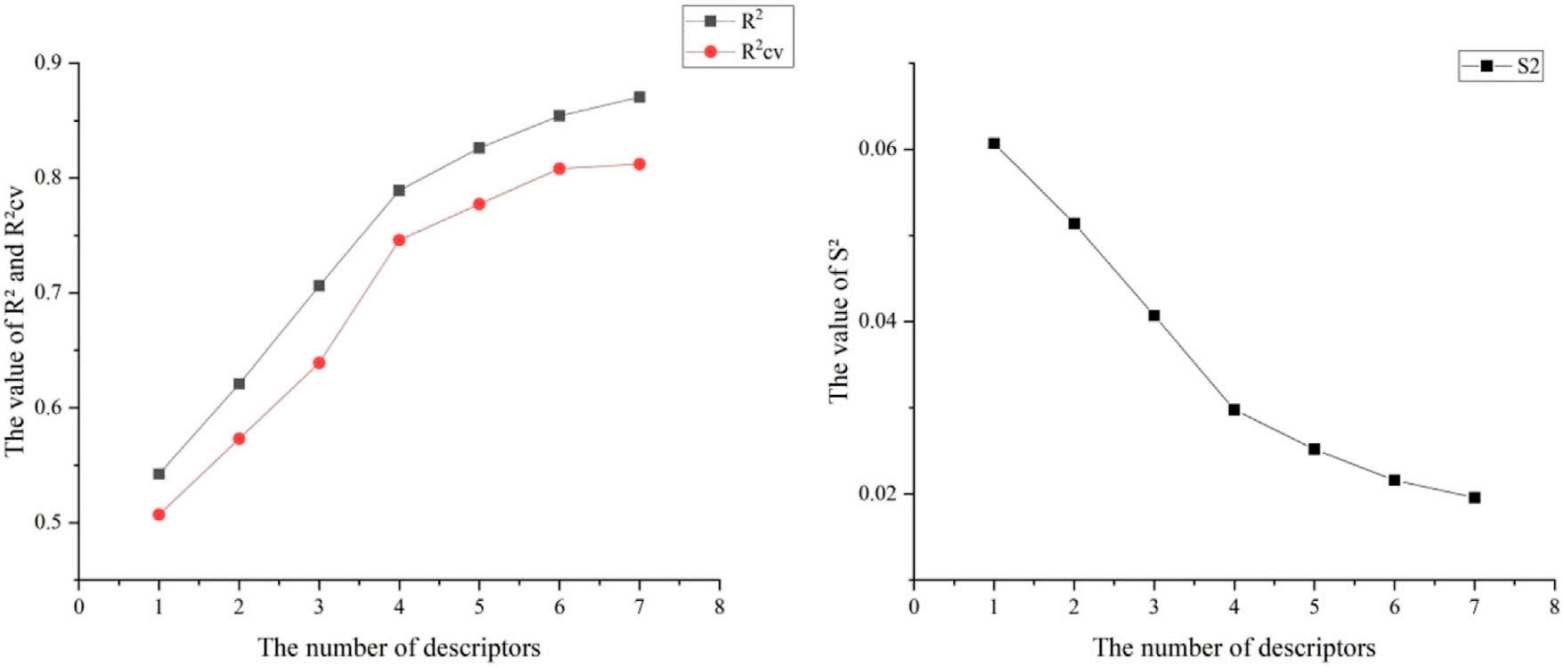
Relationship between *R* ^2^, R^2^cv, S^2^ and different numbers of descriptors.

Six hundred thirty-two descriptors for each chemical were calculated using the CODESSA program. HM was used to create a linear regression model with 1–7 descriptors. A set of descriptors most relevant to the activity of VEGFR3 inhibitors were selected. Figure 3 shows the influence of different numbers of descriptors on R^2^, R^2^cv, and S^2^. Results showed that R^2^ and R^2^cv increased with some descriptors while S^2^ decreased. As descriptors continued increasing, R^2^ and R^2^cv increased more slowly, and the trend of S^2^ decreased more slowly. As the descriptors rise to 6, the R^2^ growth trend further decreases, and the S^2^ decreasing trend is minimized. The model with five descriptors was selected as the best linear model to ensure the fitting ability. The names of these descriptors are shown in Table 2.

**TABLE 2:** Details of the five selected descriptors.

Symbol	Physical-chemical meaning	Coefficient	T-test
MREB	Max resonance energy for a C-H bond	−4.0867e+00	−6.8381
NN	Number of N atoms	6.6215e-02	2.9039
YZS/YZR	YZ Shadow/YZ Rectangle	6.3779e+00	6.2720
MPCO(ZPC)	Min partial charge for a O atom [Zefirov’s PC]	3.7446e+00	4.6862
MSEC	Min atomic state energy for a C atom	−1.0425e-01	−3.0568

**FIGURE 4:**
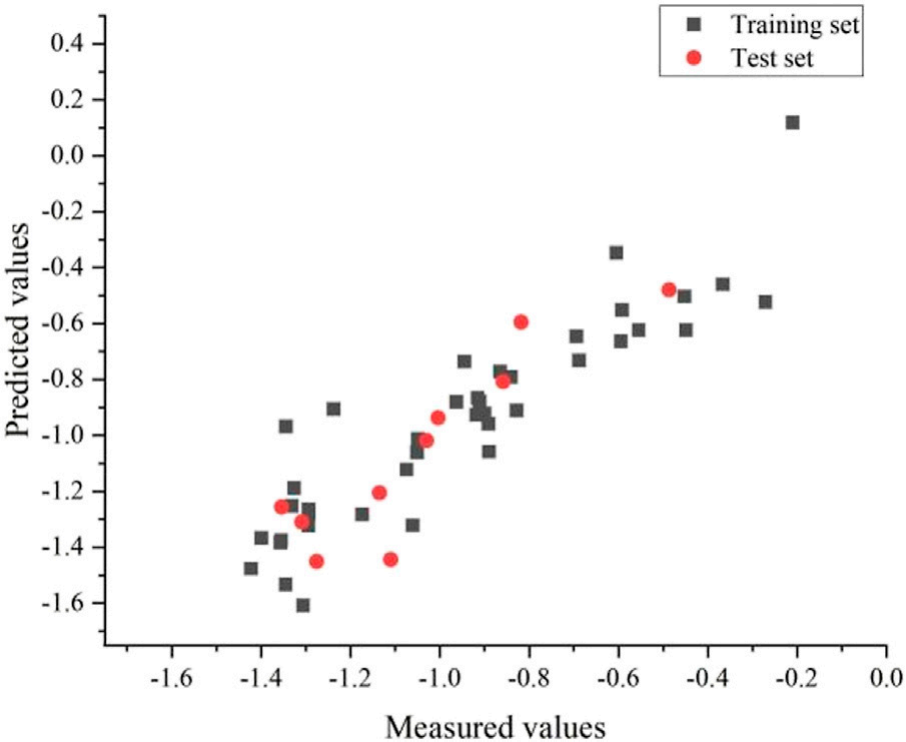
Plot of measured and predicted log(IC50) by HM.

It is difficult to accurately estimate multiple variables in linear regression models due to the influence of different factors. The correlation coefficients of the descriptors for the best linear model in this experiment are listed in Table 3. We found that all the values were below 0.8, so each descriptor existed independently. The effect of multicollinearity was excluded, proving the reliability of this linear model. Figure 4 shows the HM model.

**TABLE 3:** Correlation coefficient between descriptors.

Name	MREB	NN	YZS/YZR	MPCO(ZPC)	MSEC
MREB	1	−0.1889	−0.4369	0.4923	0.1404
NN	−0.1889	1	0.5394	−0.282	−0.2823
YZS/YZR	−0.4369	0.5394	1	−0.5592	−0.1269
MPCO(ZPC)	0.4923	−0.282	−0.5592	1	0.1121
MSEC	0.1404	−0.2823	−0.1269	0.1121	1

The best linear model had an *R* ^2^ of 0.82, a S^2^ of 0.02 and a R^2^cv of 0.77. The equation for the linear model is as follows:

$$\mathrm{Log}\left(IC50\right)=49.779-4.0867*\;MREB+6.6215*10^{-2}*\;NN+6.3779*\;YZS/YZR+3.7446*\;MPCO\left(ZPC\right)-1.0425*10^{-1}*\;MSEC$$

The following formula shows how VEGFR3 inhibitors affect activity in the following order:

*YZS/YZR* > *MREB* > *MPCO(ZPC)*> NN > MSEC.

R^2^, F-test, *t*-test, and R^2^cv values were used as criteria for model evaluation in the QSAR model procedures. However, we built a linear model with five descriptors using HM, which was insufficient to find a correlation among them, so a non-linear model was needed. We, therefore, imported the corresponding non-linear descriptors and response variables into the APS software to build such a model.

### 3.2 GEP

We imported the training and test sets into the APS software and constructed the non-linear model by the GEP algorithm using the same five descriptors used in the linear model. The functions used in the experiments are in Table 4.

**TABLE 4:** All GEP algorithm operational functions.

Parameter Name	Representation	Values
Addition	+	2
Subtraction	-	2
Multiplication	*	2
Division	—	2
Inverse	Inv	1
Cosine	Cos	1
Tangent	Tan	1

**FIGURE 5:**
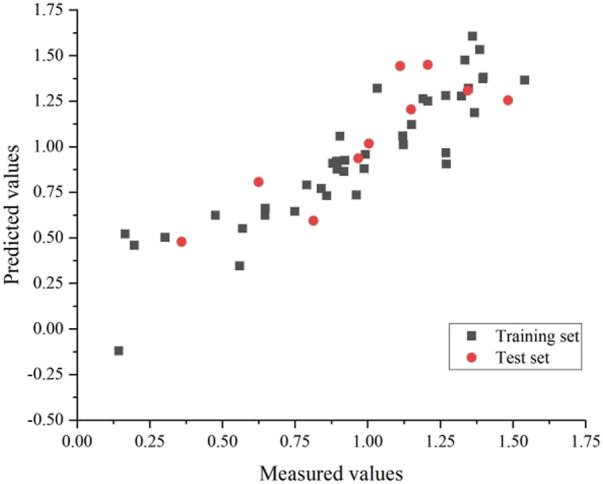
Plot of measured and predicted -log(IC_50_) by GEP.

Eventually, the training and test set’s correlation coefficients were 0.83 and 0.72, respectively, with an average error of 0.02 and 0.04. The GEP model is shown in Figure 5.

Moreover, the non-linear QSAR model through GEP was gained as follows:

$$\begin{array}{c} \mathrm{Cos}\left(\left(d\left[0\right]*d\left[2\right]\right)\right)+\mathrm{Tan}\left(\left(\left(d\left[0\right]+\mathrm{Tan}\left(\left(\left(d\left[3\right]*d\left[3\right]\right)*d\left[0\right]\right)\right)\right)-d\left[0\right]\right)\right)\\ \begin{array}{c} +\left(\left(d\left[3\right]/\mathrm{Cos}\left(\left(1/\left(\left(\left(d\left[3\right]/d\left[0\right]\right)+d\left[3\right]\right)\right)\right)\right)\right)*d\left[3\right]\right)\\ +\mathrm{Tan}\left(\mathrm{Tan}\left(\mathrm{Tan}\left(\left(\mathrm{Tan}\left(\mathrm{Cos}\left(d\left[4\right]\right)\right)/\left(d\left[1\right]/d\left[2\right]\right)\right)\right)\right)\right) \end{array}\\ +\mathrm{Tan}\left(\mathrm{Tan}\left(\mathrm{Tan}\left(\left(\mathrm{Tan}\left(\mathrm{Tan}\left(\left(d\left[0\right]+d\left[0\right]\right)\right)\right)/d\left[2\right]\right)\right)\right)\right) \end{array}$$

Where d[0], d(1), d(2), d(3), and d(4) represent MREB, the number of N atoms, YZS/YZR, MPCO(ZPC), and MSEC, respectively.

Based on the experiments of Si Y et al. (Si et al., 2022) and Chen C et al. (Chen and Si, 2021) the fitting ability of the non-linear model constructed by GEP is acceptable.

### 3.3 CoMSIA

#### 3.3.1 Statistical data

This experiment obtained the best CoMSIA model with a Q^2^ of 0.503 and an optimum component number of 2. Detailed data on the optimal CoMSIA model can be found in Table 5.

**TABLE 5:** Details of the optimal CoMSIA model.

Model	q^2^	ONC	*r* ^2^	SEE	F
CoMSIA	0.503	2	0.805	0.172	76.52
Name	S	E	H	D	A
Contribution (%)	17.8	23.7	18.8	14.5	25.3

#### 3.3.2 Model validation

**FIGURE 6:**
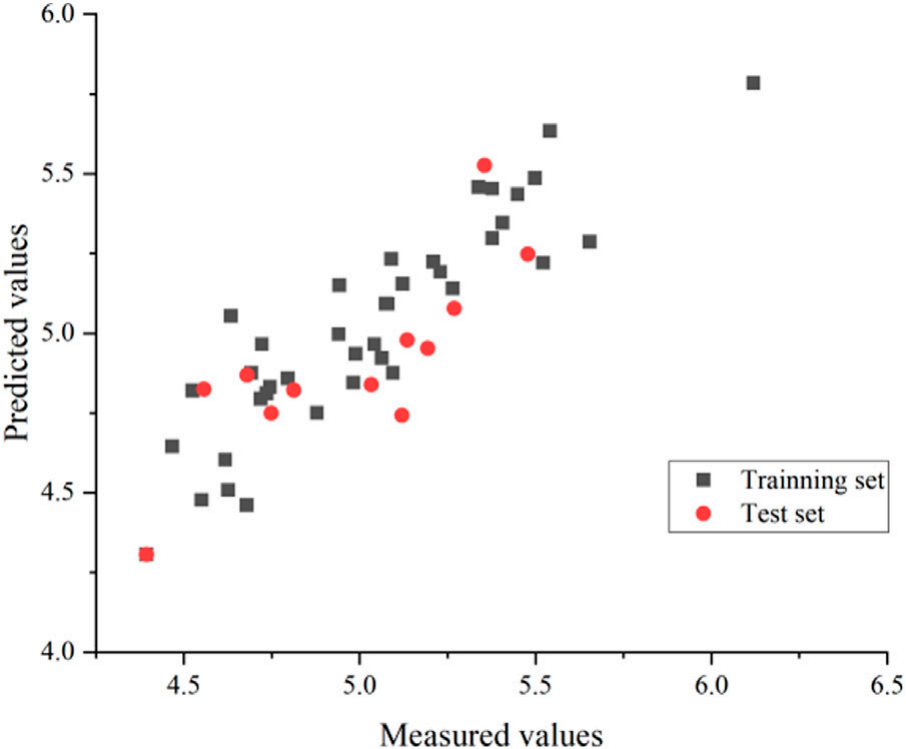
CoMSIA model predicted activity values compared with experimental values.

An external validation formula was used to validate the model in this experiment to verify whether the CoMSIA model was qualified. The external validation formula yielded a value of 0.63, which was more significant than 0.5, indicating that the model was robust and statistically had excellent predictive power. Also, we substituted all compounds into the model, and it can be seen from Figure 6 that the model’s predictive ability is reliable.

#### 3.3.3 Contour maps

The availability of contour plots is one advantage of the CoMSIA model. Because different groups have different effects on drug activity due to different binding sites in various molecular force fields, contour plots offer a detailed view of these effects. Therefore, drugs with better activity and performance in research and development can be designed according to the contour map (Li et al., 2012; Mao et al., 2012).

**FIGURE 7:**
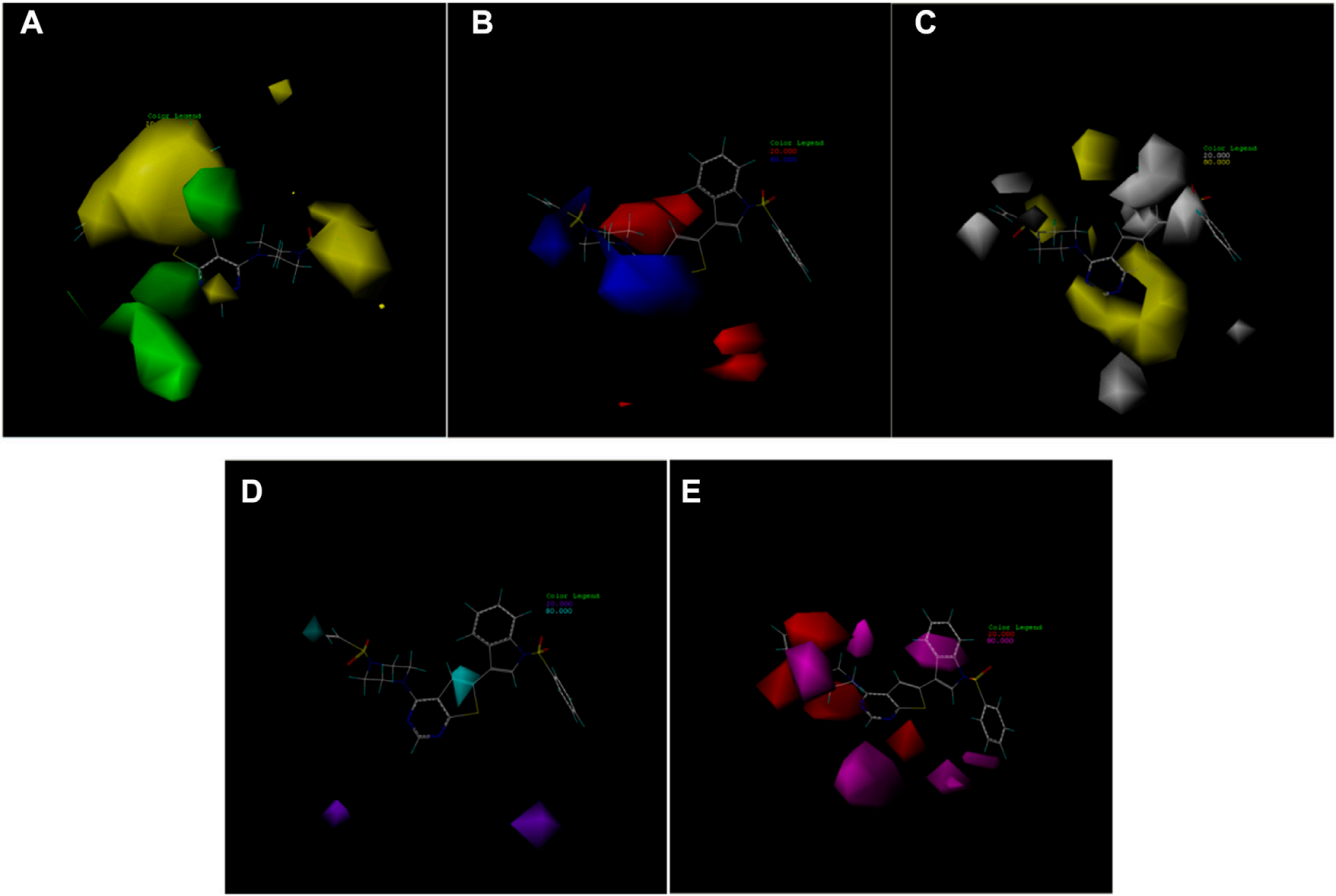
Contour map of optimal compound 14.**(A)**In the steric field, green represents favorable and yellow represents unfavorable.**(B)**In the electrostatic field, blue represents positive electric field and red represents negative electric field.**(C)**In the hydrophobic field, yellow represents favorable and white represents unfavorable.**(D)**Favorable (cyan) and unfavorable (purple) hydrogen bond donor fields.**(E)**Favorable (magenta) and unfavorable (red) hydrogen bond acceptor fields.

In this trial, according to compound 14 with the highest IC50 value, the contours of the potential spatial field, electrostatic field, hydrophobic field, hydrogen bond donor field, and hydrogen bond acceptor field of the CoMSIA model were constructed respectively (Figure 7). The largest among them is produced by the hydrogen bond acceptor field, followed by the electrostatic field. As contour plots show that the hydrogen bond acceptor field cannot be directly added to the compound structure, this study provides extensive information on the electrostatic field, which is the second contribution. The electrostatic field is observed when an observer rests relative to a charge whose charge does not change with time. It is a unique form of matter existing in the space around an electric charge, and its fundamental characteristic is the forceful action on the fixed charge placed in it.

### 3.4 Design of new compounds and the prediction of their activity

In the 2D-QSAR experimental results of VEGFR3 inhibitors, the most influential descriptor of drug activity of the compounds was YZS/YZR. Therefore, the CoMSIA model contour map and the descriptor “YZ Shadow/YZ Rectangle” should be considered in developing novel drugs.

The YZS/YZR is computed as follows (University of Florida, 2001):

$$S_{k}=1/2\underset{\left(3\right)}{\oint }\left(vd\rho -\rho dv\right)$$

YZS/YZR means the C - the contour of molecule projection on the plane defined by two principal axes of the molecule (k = XY, XZ, or YZ). Its positive coefficient indicates that the activity of VEGFR3 inhibitors will increase with increasing YZS/YZR. As a result, while designing novel compounds, the matrix area of the compound is increased per the contour electrostatic field diagram. At the same time, the reactive group at the corresponding site is added to increase the YZS/YZR.

Finally, we designed 100 novel VEGFR3 inhibitors using compound 14 as a template. The IC50 value of these 100 new compounds was predicted using the CoMSIA model. The ten compounds with the best activity are shown in Table 6. To verify their potential as anti-retinoblastoma drugs, we performed docking experiments.

**TABLE 6:** New compounds designed and their predicted values.

Name	Predictive value
14	3.967
14.a	4.83
14.b	4.83
14.c	4.704
14.d	4.685
14.e	4.682
14.f	4.674
14.g	4.559
14.h	4.554
14.i	4.542
14.j	4.533

### 3.5 Molecular docking experiment

**FIGURE 8:**
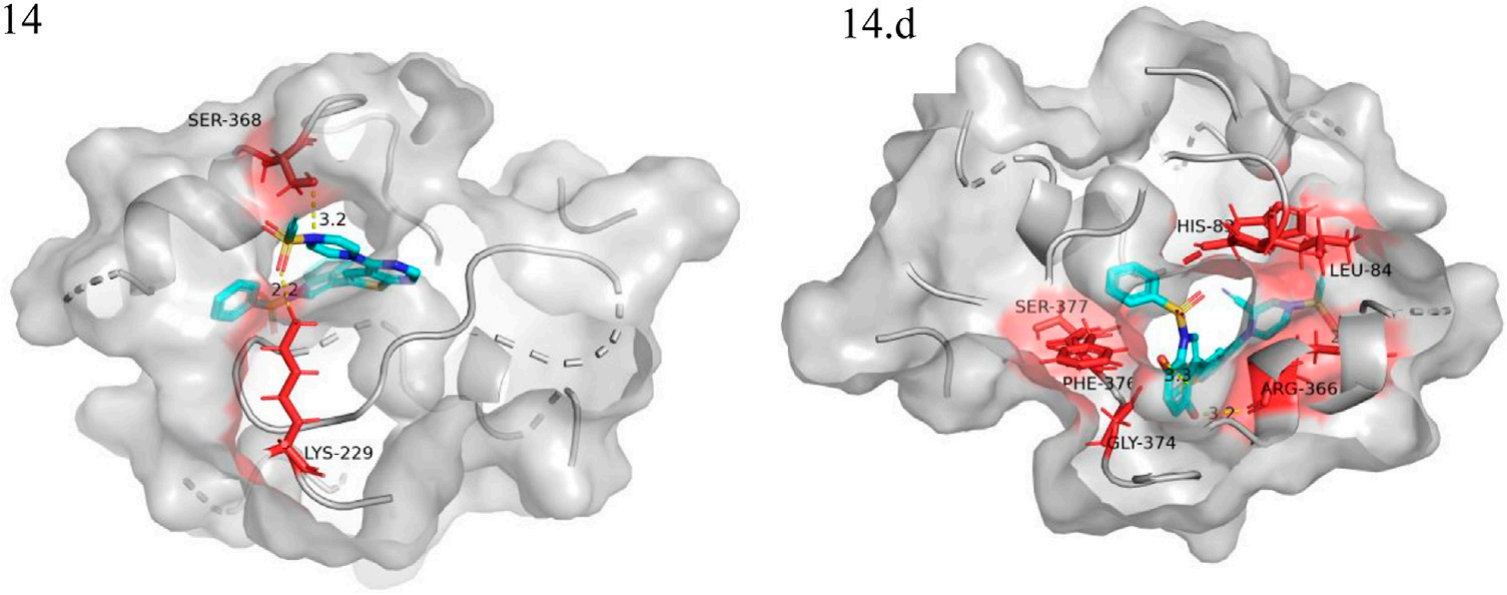
Docking experiments of compoud 14, 14.d with retinoblastoma targets (VEGFR3 homology model in the PDB format can be found in the Additional files).

We performed small molecule compound docking experiments using the SYBYL package to verify the effectiveness of the compounds newly designed in this experiment on retinoblastoma-related targets. The newly-designed compounds were imported into Sybyl software for structural optimization. The energy was converged to 0.01kcal/(mol*Å) using the Tripos force field and Gasteigera-Huckel charge (Chen et al., 2022b). The lowest energy conformation obtained was used as a small molecule ligand to dock with the VEGFR3 homology model. The higher the total score, the better the drug molecule was bound to the protein. Compound 14 and the 10 compounds with the highest predicted value were used as ligands for docking experiments based on the VEGFR3 homology model. Among them compound 14.a possessed the highest predictive value, with a crossover docking ability. On balance, compound 14.d had the highest predictive value and the most robust docking ability and had the most potential as an anti-cancer drug for retinoblastoma. Figure 8 shows the docking results of compounds 14 and 14.d (yellow dashed lines are hydrogen bonds).

## 4 Discussion

We carefully examined descriptors to understand the factors that influence IC50 deeply. The definition of Max resonance energy for a C-H bond (MREB) is given as (University of Florida, 2001).

$$E_{R}AB=\underset{\mu \epsilon A}{\sum }\underset{v\epsilon B}{\sum }P_{\mu v}\beta_{\mu v}$$

Here, A is given atomic species, B is another atomic species, Pμv is density matrix elements over atomic basis {μv.}, βμv is resonance integrals on atomic basis {μv.}. The larger the density matrix elements over atomic basis {μv.} and resonance integrals on atomic basis {μv.}, the greater the value of MREB. Because MREB is negatively correlated with IC50, the descriptor’s value is lower, while the IC50 value is better.

The calculation method of Min partial charge for an O atom [Zefirov’s PC] is as follows (University of Florida, 2001):

$$Q_{\min}=\min\left(Q^{-}\right)$$

Min partial charge for an O atom [Zefirov’s PC], the electrostatic parameter is associated with the electronegativity of the oxygen that is higher than the electronegativity of carbon, causing electrons to spend more time around the oxygen (O) atom, giving it a partial negative charge. In contrast, the carbon will become partially favourable. This parameter indicates the importance of the presence of the O atom in specific positions in the molecule. The descriptor’s value is higher, while the IC50 value is better.

The smaller its value, the higher the activity of the VEGFR3 inhibitor because the correlation coefficient of the Min atomic state energy for a C atom is negative.

The number of N atoms represents the molecular composition of the compound. Additional N atoms imply that the structure contains more NO2 or NH2 groups. This compound has higher activity because the structure can combine an H-bond with the target. The positive coefficient indicates that the higher the value of the Number of N atoms, the higher the activity of the VEGFR3 inhibitors.

The oxygen atom on 1-(ethenesulfonyl) piperazine was docked to the fragment LYS229 in the template compound 14 docking results, and the nitrogen atom was successfully docked to SER368. Hydrogen bonds could be formed with multiple fragments in compound 14.d docking result, and the compound had higher activity than the template compound.

This experiment had the advantage of using a hybrid 2D, 3D-QSAR drug design model. They enabled the consideration of the effects of groups at various positions in 3D-QSAR and the effect of descriptors on the drugs through 2D-QSAR. The activity of the designed compounds was significantly increased and verified in molecular docking experiments, which guided the design of new drugs. The experiments did not fully consider the influence of other descriptors on the development of novel drugs, which is a direction for further research.

## 5 Conclusion

Linear and non-linear QSAR models were built using heuristic methods and GEP algorithms. The non-linear model of GEP had a more stable predictive ability than the linear model of HM. Nevertheless, the effect of the three-dimensional conformation of the molecule failed to account for the two-dimensional conformational model. Hence, using the CoMSIA approach, we created a 3D-QSAR model with a high Q^2^ (0.503), R^2^ (0.805), and low SSE (0.172). Finally, we designed 100 new compounds by taking advantage of the most influential descriptors YZS/YZR in the 2D-QSAR model and the electrostatic fields that contributed prominently in the 3D-QSAR model and predicted their activities based on the CoMSIA model. The 10 compounds with the highest activity were selected for small molecule docking experiments. Compound 14.d had good drug activity and docking ability. Consequently, this study provides an innovative approach to developing anti-cancer drugs for treating retinoblastoma.

## Data Availability

The raw data supporting the conclusion of this article will be made available by the authors, without undue reservation.

## Abbreviations

QSAR, quantitative structure—activity relationship; VEGFR3, vascular endothelial growth factor receptor 3; HM, heuristic method; GEP, gene expression programming; CoMSIA, comparative molecular similarity indices analysis; MREB, max resonance energy for a C-H bond; NN, number of N atoms; YZS/YZR, YZ Shadow/YZ Rectangle; MPCO(ZPC), min partial charge for a O atom [Zefirov’s PC]; MSEC, min atomic state energy for a C atom.

## References

[B1] Ai Y. Wang S. T. Tang C. Sun P. H. Song F. J. (2011). 3D-QSAR and docking studies on pyridopyrazinones as BRAF inhibitors. Med. Chem. Res. 20, 1298–1317. 10.1007/s00044-010-9468-1

[B2] Ajala A. O. Okoro C. O. (2011). CoMFA and CoMSIA studies on fluorinated hexahydropyrimidine derivatives. Bioorg. Med. Chem. Lett. 21 (24), 7392–7398. 10.1016/j.bmcl.2011.10.008 22056745

[B3] Alam A. Blanc I. Gueguen-Dorbes G. Duclos O. Bonnin J. Barron P. (2012). SAR131675, a potent and selective VEGFR-3-TK inhibitor with antilymphangiogenic, antitumoral, and antimetastatic activities. Mol. Cancer Ther. 11 (8), 1637–1649. 10.1158/1535-7163.MCT-11-0866-T 22584122

[B4] Algorithms G. (1992). Computer programs that" evolve" in ways that resemble natural selection can solve complex problems even their creators do not fully understand[J]. Holland in Scientific American, 66–72.

[B5] Cao C. Lin Y. (2003). Correlation between the glass transition temperatures and repeating unit structure for high molecular weight polymers. J. Chem. Inf. Comput. Sci. 43 (2), 643–650. 10.1021/ci0202990 12653533

[B6] Chang Y. W. Su C. M. Su Y. H. Ho Y. S. Lai H. H. Chen H. A. (2014). Novel peptides suppress VEGFR-3 activity and antagonize VEGFR-3-mediated oncogenic effects. Oncotarget 5 (11), 3823–3835. 10.18632/oncotarget.1709 25003617 PMC4116523

[B7] Chen C. Si H. (2021). QSAR models of Celecoxib analogues and derivatives as COX-2 inhibitor to predict their anti-inflammatory effect[J]. Cancer Cell. 33 (2021), 827–835.

[B8] Chen Y. Ma K. Si H. Duan Y. Zhai H. (2022). Network Pharmacology integrated molecular docking to reveal the autism and mechanism of baohewan heshiwei wen dan tang. Curr. Pharm. Des. 28 (39), 3231–3241. 10.2174/1381612828666220926095922 36165527

[B9] Chen Y. Ma K. Xu P. Si H. Duan Y. Zhai H. (2022). Design and screening of new lead compounds for autism based on QSAR model and molecular docking studies. J. Mol. 27 (21), 7285. 10.3390/molecules27217285 PMC9657114 36364109

[B10] Cherkasov A. Muratov E. N. Fourches D. Varnek A. Baskin II Cronin M. (2014). QSAR modeling: Where have you been? Where are you going to. J. Med. Chem. 57 (12), 4977–5010. 10.1021/jm4004285 24351051 PMC4074254

[B11] Cramer R. D. Patterson D. E. Bunce J. D. (1988). Comparative molecular field analysis (CoMFA). 1. Effect of shape on binding of steroids to carrier proteins. J. Am. Chem. Soc. 110 (18), 5959–5967. 10.1021/ja00226a005 22148765

[B12] Dhami A. Bansal A. Khetan V. (2017). Retinoblastoma: An overview of modern management. Nepal J. Ophthalmol. 9 (18), 1–12. 10.3126/nepjoph.v9i1.17524 29022948

[B13] Gharagheizi F. Ilani-Kashkouli P. Farahani N. Mohammadi A. H. (2012). Gene expression programming strategy for estimation of flash point temperature of non-electrolyte organic compounds. Fluid Phase Equilibria 329, 71–77. 10.1016/j.fluid.2012.05.015

[B14] Hamada K. Oike Y. Takakura N. Ito Y. Jussila L. Dumont D. J. (2000). VEGF-C signaling pathways through VEGFR-2 and VEGFR-3 in vasculoangiogenesis and hematopoiesis. Blood 96 (12), 3793–3800. 10.1182/blood.v96.12.3793 11090062

[B15] Holland J. H. (1992). Genetic algorithms. Sci. Am. 267 (1), 66–72. 10.1038/scientificamerican0792-66 1411454

[B16] HyperChem. 4.0 (1994). Hypercube.

[B17] Jiang X. Zhang Q. L. Liu T. G. Zhao W. P. Yang M. Wang L. N. (2019). Evaluation of local injection of bevacizumab against triple-negative breast cancer xenograft tumors. Curr. Pharm. Des. 25 (8), 862–870. 10.2174/1381612825666190306164157 30848190

[B18] Jiang Y. Z. Liu Y. Xiao Y. Hu X. Jiang L. Zuo W. J. (2021). Molecular subtyping and genomic profiling expand precision medicine in refractory metastatic triple-negative breast cancer: The FUTURE trial. Cell. Res. 31 (2), 178–186. 10.1038/s41422-020-0375-9 32719455 PMC8027015

[B19] Kaydani H. Mohebbi A. Eftekhari M. (2014). Permeability estimation in heterogeneous oil reservoirs by multi-gene genetic programming algorithm. J. Petroleum Sci. Eng. 123, 201–206. 10.1016/j.petrol.2014.07.035

[B20] Kaydani H. Mohebbi A. Eftekhari M. (2014). Permeability estimation in heterogeneous oil reservoirs by multi-gene genetic programming algorithm. J. Petroleum Sci. Eng. 123, 201–206. 10.1016/j.petrol.2014.07.035

[B21] Kirkin V. Thiele W. Baumann P. Mazitschek R. Rohde K. Fellbrich G. (2004). MAZ51, an indolinone that inhibits endothelial cell and tumor cell growth*in vitro*, suppresses tumor growth*in vivo* . Int. J. Cancer 112 (6), 986–993. 10.1002/ijc.20509 15386354

[B22] Klebe G. Abraham U. Mietzner T. (1994). Molecular similarity indices in a comparative analysis (CoMSIA) of drug molecules to correlate and predict their biological activity. J. Med. Chem. 37 (24), 4130–4146. 10.1021/jm00050a010 7990113

[B23] Li H. M. Dong Z. P. Wang Q. Y. Liu L. X. Li B. X. Ma X. N. (2017). De novo computational design for development of a peptide ligand oriented to VEGFR-3 with high affinity and long circulation. Mol. Pharm. 14 (7), 2236–2244. 10.1021/acs.molpharmaceut.7b00070 28506066

[B24] Li X. Ye L. Wang X. Liu H. Zhu Y. (2012). Combined 3D-QSAR, molecular docking and molecular dynamics study on thyroid hormone activity of hydroxylated polybrominated diphenyl ethers to thyroid receptors β. Toxicol. Appl. Pharmacol. 265 (3), 300–307. 10.1016/j.taap.2012.08.030 22982074

[B25] Li Y. Wang Y. H. Yang L. Zhang S. W. Liu C. H. Yang S. L. (2005). Comparison of steroid substrates and inhibitors of P-glycoprotein by 3D-QSAR analysis. J. Mol. Struct. 733 (1-3), 111–118. 10.1016/j.molstruc.2004.08.012

[B26] Li Y. Yang G. Zhang J. Tang P. Yang C. Wang G. (2021). Discovery, synthesis, and evaluation of highly selective vascular endothelial growth factor receptor 3 (VEGFR3) inhibitor for the potential treatment of metastatic triple-negative breast cancer. J. Med. Chem. 64 (16), 12022–12048. 10.1021/acs.jmedchem.1c00678 34351741

[B27] Luan M. Si H. (2022). Novel hypoxia features with appealing implications in discriminating the prognosis, immune escape and drug responses of 947 hepatocellular carcinoma patients. Transl. Cancer Res. 11 (7), 2097–2121. 10.21037/tcr-22-253 35966318 PMC9372209

[B28] Mao Y. Li Y. Hao M. Zhang S. Ai C. (2012). Docking, molecular dynamics and quantitative structure-activity relationship studies for HEPTs and DABOs as HIV-1 reverse transcriptase inhibitors. J. Mol. Model. 18, 2185–2198. 10.1007/s00894-011-1236-8 21947448

[B29] Melincovici C. S. Boşca A. B. Şuşman S. Mărginean M. Mihu C. Istrate M. (2018). Vascular endothelial growth factor (VEGF) - key factor in normal and pathological angiogenesis. Rom. J. Morphol. Embryol. 59 (2), 455–467. 30173249

[B30] Mouchlis V. D. Melagraki G. Mavromoustakos T. Kollias G. Afantitis A. (2012). Molecular modeling on pyrimidine-urea inhibitors of TNF-α production: An integrated approach using a combination of molecular docking, classification techniques, and 3D-QSAR CoMSIA. J. Chem. Inf. Model. 52 (3), 711–723. 10.1021/ci200579f 22360289

[B31] Patel P. D. Patel M. R. Kaushik-Basu N. Talele T. T. (2008). 3D QSAR and molecular docking studies of benzimidazole derivatives as hepatitis C virus NS5B polymerase inhibitors. J. Chem. Inf. Model. 48 (1), 42–55. 10.1021/ci700266z 18076152

[B32] Pham D. Karaboga D. (2012). Intelligent optimisation techniques: Genetic algorithms, tabu search, simulated annealing and neural networks[M]. Springer Science & Business Media.

[B33] Ravi V. Sanford E. M. Wang W. L. Ross J. S. Ramesh N. Futreal A. (2016). Antitumor response of VEGFR2-and VEGFR3-amplified angiosarcoma to pazopanib. J. Natl. Compr. Canc Netw. 14 (5), 499–502. 10.6004/jnccn.2016.0058 27160228

[B34] Roy K. Kar S. Das R. N. (2015). Understanding the basics of QSAR for applications in pharmaceutical sciences and risk assessment[M]. Academic Press.

[B35] Santos-Filho O. A. Hopfinger A. J. (2001). A search for sources of drug resistance by the 4D-QSAR analysis of a set of antimalarial dihydrofolate reductase inhibitors. J. Comput. Aided Mol. Des. 15 (1), 1–12. 10.1023/a:1011152818340 11217916

[B36] Si Y. Ma K. Hu Y. Si H. Zhai H. (2022). QSAR model study of 2,3,4,5-tetrahydro-1H-pyrido[4,3-b]indole of cystic-brosis-transmembrane conductance-regulator gene potentiators. Lett. Drug Des. Discov. 19 (4), 269–278. 10.2174/1570180818666211022142920

[B37] Teodorescu L. Sherwood D. (2008). High energy physics event selection with gene expression programming. Comput. Phys. Commun. 178 (6), 409–419. 10.1016/j.cpc.2007.10.003

[B38] University of Florida (2001). Shadow areas of a molecule. Available at: http://www.codessa-pro.com/descriptors/geom/sam.htm.

[B39] Wu Q. Sun X. Zheng G. (2018). VEGF overexpression is associated with optic nerve involvement and differentiation of retinoblastoma: A PRISMA-compliant meta-analysis. Med. Baltim. 97 (51), e13753. 10.1097/MD.0000000000013753 PMC6319877 30572521

[B40] Yan W. Lin G. Zhang R. Liang Z. Wu W. (2020). Studies on the bioactivities and molecular mechanism of antioxidant peptides by 3D-QSAR, *in vitro* evaluation and molecular dynamic simulations. Food & Funct. 11 (4), 3043–3052. 10.1039/c9fo03018b 32190865

[B41] Yang Y. Qin J. Liu H. Yao X. (2011). Molecular dynamics simulation, free energy calculation and structure-based 3D-QSAR studies of B-RAF kinase inhibitors. J. Chem. Inf. Model. 51 (3), 680–692. 10.1021/ci100427j 21338122

[B42] Yu Z. Li X. Ge C. Si H. Cui L. Gao H. (2015). 3D-QSAR modeling and molecular docking study on Mer kinase inhibitors of pyridine-substituted pyrimidines. Mol. Divers. 19, 135–147. 10.1007/s11030-014-9556-0 25355276

[B43] Zeidman I. Copeland B. E. Warren S. (1955). Experimental studies on the spread of cancer in the lymphatic system. II. Absence of a lymphatic supply in carcinoma. Cancer 8 (1), 123–127. 10.1002/1097-0142(1955)8:1<123::aid-cncr2820080116>3.0.co;2-a 13231041

[B44] Zhang L. Chen J. Gao C. Liu C. Xu K. (2018). An efficient model for auxiliary diagnosis of hepatocellular carcinoma based on gene expression programming. Med. Biol. Eng. Comput. 56, 1771–1779. 10.1007/s11517-018-1811-6 29546505

